# Tumor-specific usage of alternative transcription start sites in colorectal cancer identified by genome-wide exon array analysis

**DOI:** 10.1186/1471-2164-12-505

**Published:** 2011-10-14

**Authors:** Kasper Thorsen, Troels Schepeler, Bodil Øster, Mads H Rasmussen, Søren Vang, Kai Wang, Kristian Q Hansen, Philippe Lamy, Jakob Skou Pedersen, Asger Eller, Francisco Mansilla, Kirsti Laurila, Carsten Wiuf, Søren Laurberg, Lars Dyrskjøt, Torben F Ørntoft, Claus L Andersen

**Affiliations:** 1 Department of Molecular Medicine, Aarhus University Hospital, Skejby, 8200 Aarhus N, Denmark; 2 Department of Biostatistics and Computational Biology, Dana-Farber Cancer Institute, Harvard School of Public Health, Harvard University, Boston, MA 02115, USA; 3 Department of Radiology, Harvard Medical School, Harvard University, Boston, MA 02115, USA; 4 Bioinformatics Research Centre (BiRC), Aarhus University, 8000 Aarhus C, Denmark; 5 Department of Signal Processing, Tampere University of Technology, P.O. Box 527, FI-33101 Tampere, Finland; 6 Department of Surgery P, Aarhus University Hospital, THG, 8000 Aarhus C, Denmark

## Abstract

**Background:**

Approximately half of all human genes use alternative transcription start sites (TSSs) to control mRNA levels and broaden the transcriptional output in healthy tissues. Aberrant expression patterns promoting carcinogenesis, however, may arise from alternative promoter usage.

**Results:**

By profiling 108 colorectal samples using exon arrays, we identified nine genes (*TCF12, OSBPL1A, TRAK1, ANK3, CHEK1, UGP2, LMO7, ACSL5*, and *SCIN*) showing tumor-specific alternative TSS usage in both adenoma and cancer samples relative to normal mucosa. Analysis of independent exon array data sets corroborated these findings. Additionally, we confirmed the observed patterns for selected mRNAs using quantitative real-time reverse-transcription PCR. Interestingly, for some of the genes, the tumor-specific TSS usage was not restricted to colorectal cancer. A comprehensive survey of the nine genes in lung, bladder, liver, prostate, gastric, and brain cancer revealed significantly altered mRNA isoform ratios for *CHEK1, OSBPL1A*, and *TCF12 *in a subset of these cancer types.

To identify the mechanism responsible for the shift in alternative TSS usage, we antagonized the Wnt-signaling pathway in DLD1 and Ls174T colorectal cancer cell lines, which remarkably led to a shift in the preferred TSS for both *OSBPL1A *and *TRAK1*. This indicated a regulatory role of the Wnt pathway in selecting TSS, possibly also involving TP53 and SOX9, as their transcription binding sites were enriched in the promoters of the tumor preferred isoforms together with their mRNA levels being increased in tumor samples.

Finally, to evaluate the prognostic impact of the altered TSS usage, immunohistochemistry was used to show deregulation of the total protein levels of both TCF12 and OSBPL1A, corresponding to the mRNA levels observed. Furthermore, the level of nuclear TCF12 had a significant correlation to progression free survival in a cohort of 248 stage II colorectal cancer samples.

**Conclusions:**

Alternative TSS usage in colorectal adenoma and cancer samples has been shown for nine genes, and *OSBPL1A *and *TRAK1 *were found to be regulated *in vitro *by Wnt signaling. TCF12 protein expression was upregulated in cancer samples and correlated with progression free survival.

## Background

Colorectal cancer (CRC) is a leading cause of cancer mortality with more than 600,000 deaths per year globally [1]. CRC can be divided into two major subgroups: microsatellite stable (MSS) and microsatellite unstable cancers (MSI), the latter being characterized by a defective mismatch repair system, which leads to mutations in microsatellite repeat regions [2]. The two subgroups also show differences in transcriptional profiles and clinical disease course [3]. A key event in the transformation of colonic epithelial cells is the activation of the Wnt-signaling pathway, which is observed in the vast majority of colorectal tumors [4]. Following activation of the Wnt pathway, β-catenin accumulates in the nucleus where it interacts with members of the TCF/LEF transcription factors, such as TCF1 (gene symbol *TCF7*) and TCF4 (gene symbol *TCF7L2*) leading to expression of target genes including MYC, SOX9 and Cyclin D1. Furthermore, it has previously been shown that Wnt signaling also plays a regulatory role in alternative splicing [5,6].

The generation of proteome diversity from a rather limited number of genes is primarily due to alternative splicing and alternative promoter usage, the latter leading to multiple transcripts from the same gene with different transcription start sites (TSSs). It has been estimated that 30-50% of all human genes have multiple promoters [7-9], and among the genes with experimentally well described alternative promoters are several cancer related genes such as *MYC *[10], *TP53 *[11] and *BRCA1 *[12]. Genome-wide predictive analysis has shown that alternative promoters are overrepresented in genes involved in development and transcriptional control, whereas genes with only a single promoter are more frequently involved in general cellular processes [9]. Alternative TSS can have crucial impact on both the transcriptional level and stability of the transcript as well as the function and cellular localization of the translated protein [8]. An example of a transcript with directly opposite effects is the transcription factor TCF1 where the use of alternative promoters results in the formation of two isoforms with different β-catenin binding capability. While the long isoform of TCF1 interacts with β-catenin and stimulates transcription of Wnt-target genes, the short isoform, unable to bind β-catenin but with the DNA binding domain intact, acts as a dominant negative regulator of β-catenin mediated Wnt signaling [13].

In the present study, a genome-wide search revealed nine genes with tumor-specific TSS usage in neoplastic colorectal tissue samples. For some of the genes, the tumor-specific TSS usage was not restricted to CRC, but was observed in other cancer types as well. The use of alternative TSSs in *OSBPL1A *and *TRAK1 *was found to be regulated by the Wnt pathway and also observed in gastric, prostate and brain tumors. Protein levels of OSBPL1A and TCF12 were found to be deregulated in colorectal tumors and the protein expression of TCF12 correlated with progression free survival of stage II CRC.

## Results

### Identification of differential transcription start site usage

To investigate whether the use of alternative TSSs was associated with tumor development, normal colorectal mucosa (n = 24), colorectal adenoma (n = 49) and CRC samples (n = 35) were profiled using the Affymetrix Human Exon 1.0 ST Array. The Refseq database (hg18) was queried for genes with two or more known transcripts with different TSSs. In total, 2176 protein-coding genes containing multiple TSSs were identified and matched to exon array core transcript clusters by gene symbol of which 2036 genes had available exon expression data. Genes, containing exons potentially involved in tumor associated alternative TSS usage or alternative splicing, were identified using a splicing ANOVA approach. Additional filtering criteria (see methods) were used to select 156 candidate genes for manual inspection in a genomic context. The manual curation indicated tumor associated alternative TSS usage in nine (*TCF12, OSBPL1A, TRAK1, ANK3, CHEK1, UGP2, LMO7, ACSL5 *and *SCIN*) of the 156 genes. To confirm these findings, two independent CRC sample sets (covering a total of 18 normal mucosa, six adenomas and 28 CRC samples) were investigated. All nine genes showed expression profiles consistent with tumor-specific alternative TSS usage in at least one of the independent validation sets.

### qRT-PCR validation of alternative TSS candidates

**Figure 1:**
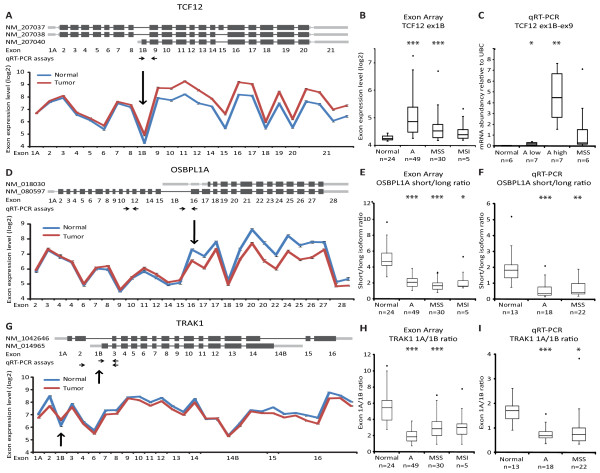
**Expression of *TCF12, OSBPL1A *and *TRAK1 *isoforms in colorectal neoplasia**. A, Exon structure and exon array expression of *TCF12 *in normal mucosa (n = 24) and tumor (n = 84) samples. Error bars represent SE. B, Exon array expression levels of the alternative start exon (1B) in mucosa samples, adenomas, and carcinomas (MSS and MSI). C, qRT-PCR validation of exon 1B expression, primer position is indicated in A. D, Exon structure and exon array expression of *OSBPL1A *as in A. E, Exon array isoform ratio of *OSBPL1A *as in B. F, qRT-PCR validation of *OSBPL1A *isoform ratio. G, Exon structure and exon array expression of *TRAK1 *as in A. H, Exon array isoform ratio of *TRAK1 *as in B. I, qRT-PCR validation of *TRAK1 *isoform ratio. * = p < 0.05, ** = p < 0.01 and *** = p < 0.001.

Three candidate genes (*TCF12, OSBPL1A *and *TRAK1*) with different expression characteristics were chosen for technical validation with quantitative real-time reverse-transcription PCR (qRT-PCR) on a subset of the samples used for exon array analysis. Transcription factor 12 (TCF12), a member of the basic helix-loop-helix E-protein family with an E-box consensus binding site, exists as a long and a short variant. According to the exon array data, the long variant was expressed in both normal and tumor samples at roughly the same level, whereas the short isoform was expressed in the majority of the tumor samples, but not detected above background in the normal samples (Figure 1A). This indicated that the short isoform of *TCF12 *was *de novo *synthesized in the majority of the tumor samples, especially in adenomas and MSS carcinomas (Figure 1B). Exon array data from paired normal and adenoma samples were available for 18 patients, and for five of these patients exon array expression data were available for a matched carcinoma sample as well. Increased expression of *TCF12 *exon 1B in tumor samples was seen in 21 of 23 sample pairs (p = 0.001) (Additional file 1A). For qRT-PCR validation of the shift in *TCF12 *TSS usage, six normal samples, 14 adenoma samples, divided in two groups based on expression of the short *TCF12 *isoform (high or low), and six MSS cancer samples were selected. qRT-PCR confirmed *de novo *expression of the short *TCF12 *isoform (exon 1B) in many tumor samples (Figure 1C) and showed strong correlation to the exon array data (RS = 0.82, Spearman correlation).

Oxysterol-binding protein-related protein 1 (OSBPL1A) is an intracellular lipid receptor which is a member of the oxysterol-binding protein (OSBP) family and exists as a long and a short transcript variant. The long variant was expressed at roughly the same level in normal and tumor samples, whereas the short variant was down-regulated in most tumor samples (Figure 1D). The ratio between the long and short variant was shifted significantly towards the long variant in both adenoma and cancer samples (Figure 1E). This shift was also highly significant (p = 7 × 10^-10^) in the 23 paired normal and tumor samples, where a lower ratio was observed in all 23 tumor samples (Additional file 1B). This tumor-specific isoform shift was confirmed by qRT-PCR in 53 tissue samples (13 normal, 18 adenomas and 22 MSS cancers) (Figure 1F), which showed a robust correlation between exon array and qRT-PCR data (RS = 0.52, Spearman correlation).

Finally, Trafficking kinesin-binding protein 1 (TRAK1), a kinesin-binding protein associated with mitochondria, contains two alternative start exons (exon 1A and 1B) with differential expression in normal and tumor samples (Figure 1G). The ratio was shifted towards exon 1B in both adenoma and cancer samples (Figure 1H), including all 23 tumors with matched normal samples (p = 5 × 10^-8^) (Additional file 1C). This trend was also confirmed by qRT-PCR in the same 53 tissue samples described above (Figure 1I). Again, a fine correlation between exon array and qRT-PCR data was observed (RS = 0.58). We speculated whether alternative splicing rather than alternative TSS could explain the observed findings for *TRAK1*. However, in both normal and tumor samples only one qRT-PCR product was observed for the primer set spanning the alternative *TRAK1 *exon 1B (from exon 2 to 3), with a length consistent with lack of exon 1B. This demonstrates that no transcript containing exons 2, 1B and 3 exists. By contrast PCR with primers in exon 1B and 3 produces a product. This indicates that the transcript template of the latter product starts with exon 1B, and, hence, that alternative TSS rather than alternative splicing is the mechanism generating the transcript. In conclusion, qRT-PCR confirmed the tumor associated alternative TSSs for all three candidates, demonstrating that exon array data reliably identify alternative TSS usage.

### qRT-PCR validation of alternative TSS in laser capture microdissected samples

It is well known that tissue composition changes from normal mucosa to tumor biopsies, and, potentially, the observed changes in TSS usage could reflect this rather than altered expression in the neoplastic cells. To address this question, we analyzed *TCF12, OSBPL1A *and *TRAK1 *isoform expression in laser capture microdissected (LCM) samples from six patients. From each patient, tumor and adjacent normal tissue biopsies were dissected to provide four samples per patient (normal epithelial cells, stroma from normal biopsies, CRC cells and cancer derived stroma). The short *TCF12 *isoform was expressed in half of the patients, and solely in cancer cells or, interestingly, cancer derived stroma, thus, confirming the tumor-specificity we observed in non-LCM samples (Additional file 2A). For *OSBPL1A*, a primer set measuring the long variant showed expression in half of the cancer and the cancer derived stroma samples, whereas the expression in the normal epithelium was low in all but one sample. A primer set measuring the expression of both isoforms showed low expression levels in both cancer epithelium and cancer derived stroma, whereas four of the six patients had a markedly higher expression in the normal epithelium and, for the same samples, a lower expression in the stroma from normal biopsies (Additional file 2B). *TRAK1 *exon 1A was found to be expressed in only two normal epithelial samples, whereas no expression of the tumor preferred exon 1B was observed in either fraction from the normal sample. Exon 1B was expressed in half of the cancer epithelium samples of which two also had expression of exon 1A (Additional file 2C). In summary, the experiments confirmed that the tumor associated alternative TSS usages observed for *TCF12, OSBPL1A *and *TRAK1 *were indeed due to altered expression in the carcinoma cells, and not a consequence of altered tissue composition.

### Alternative TSS use in other cancer types

**Figure 2:**
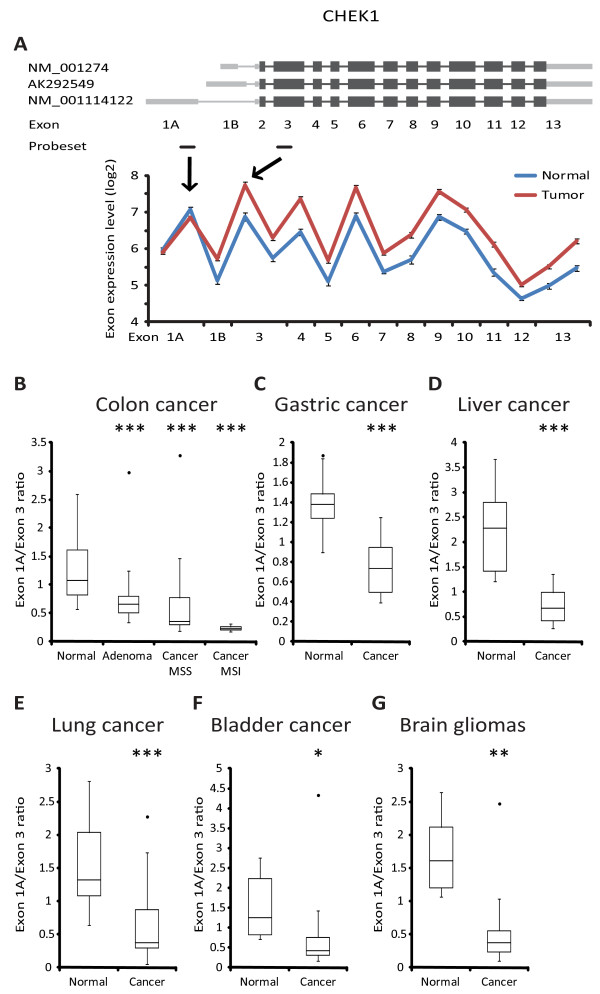
***CHEK1 *isoform expression in colorectal and other cancers**. A, Exon structure and exon array expression of *CHEK1 *in normal colorectal mucosa (n = 24) and colorectal tumor (n = 84) samples. Error bars represent SE. B, Exon array isoform ratio of *CHEK1 *in normal colorectal mucosa samples, adenomas, and carcinomas (MSS and MSI), C, expression in normal gastric and gastric cancer samples, D, expression in liver normal and cancer, E, expression in lung normal and cancer F, expression in bladder normal and cancer and, G, expression in normal brain and brain gliomas. * = p < 0.05, ** = p < 0.01 and *** = p < 0.001.

Because many of the cancer associated expression changes identified to date generally play a role in cancers from different organs, we speculated whether the collection of tumor associated alternative TSSs identified here in the context of CRC could also have relevance to other cancers. To address this question, we acquired exon array expression data from gastric, liver, lung, bladder, brain and prostate normal and cancer samples. *CHEK1 *tumor associated alternative TSSs usage, which yields transcripts differing only in their 5' untranslated region (UTR) (Figure 2A), was most widely associated with cancer. In addition to CRC, a significant change in TSS usage was found in gastric, liver, lung and bladder cancer along with brain gliomas (Figure 2B-G). Significant expression changes of the *OSBPL1A *isoforms were observed in gastric and prostate cancer and metastases from prostate cancer patients along with brain gliomas (Additional file 3A-C). For liver, lung and bladder cancer, the trend was the same although not significant (Additional file 3D-F). Isoform expression changes were also found for *TCF12 *in brain gliomas (Additional file 3G). In summary, the analysis confirmed that some of the observed tumor associated TSS usages were not restricted to CRC.

### Transcription factor binding analysis

Differences in preferred TSS could be explained by differential expression of transcription factors (TFs) controlling the activation of promoters located near the TSS of the nine selected genes. To identify TFs potentially responsible for the observed shifts in expressed isoforms, we performed TF binding site enrichment analysis in the TOUCAN2 software [14]. We chose to look at the sequence 300 bp upstream and 100 bp downstream from the TSS, which was defined as the start site of the matching Refseq sequence. Significantly overrepresented TF binding matrices were queried against the exon array expression data to identify deregulated TFs with enriched binding. Five TFs were significantly downregulated in tumor samples (p < 0.05, Benjamini-Hochberg corrected) and had enriched binding motifs near the TSS most commonly used in the normal mucosa samples (MEIS1, NR3C1, HNF4, IKZF3 and FOXO1), whereas only two TFs, TP53 and SOX9, were found to be significantly upregulated in tumor samples (p < 0.05, Benjamini-Hochberg corrected) along with having binding enrichment near the preferred TSS of the carcinoma samples, and, interestingly, SOX9 is known to be regulated by the Wnt pathway [15]. In summary, tumor-specific TF deregulation may contribute to the observed change of TSS usage.

### The Wnt pathway regulates the expression of *OSBPL1A *isoforms

**Figure 3:**
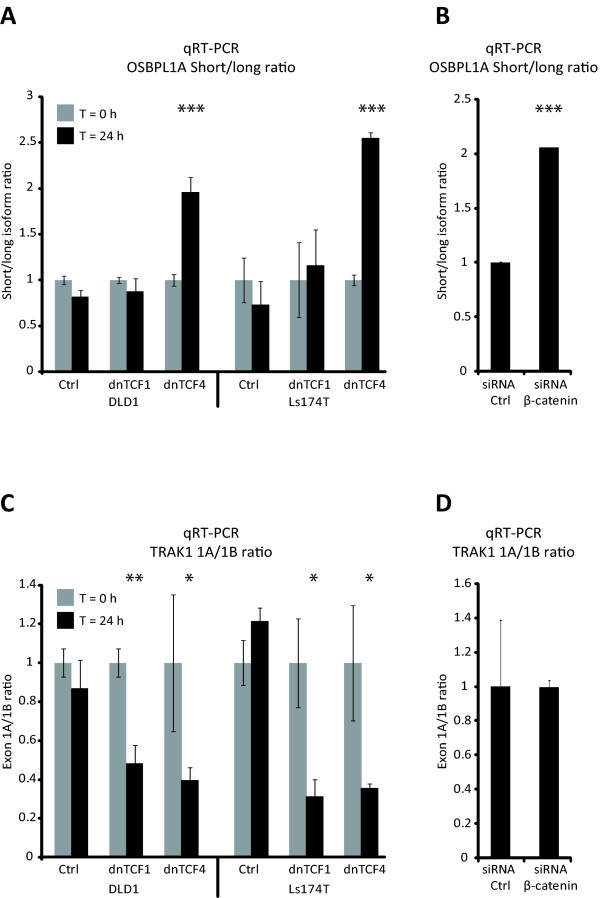
**Wnt signaling regulates isoform expression of *OSBPL1A *and *TRAK1***. A, *OSBPL1A *isoform ratio measured by qRT-PCR in DLD1 and Ls174T cells expressing either dnTCF1 or dnTCF4 to disrupt the Wnt pathway. B, *OSBPL1A *isoform ratio in DLD1 cells transfected with either 20 nM control or β-catenin siRNA. C, *TRAK1 *exon 1A/1B ratio as described in A, D, *TRAK1 *exon 1A/1B ratio as described in B. * = p < 0.05, ** = p < 0.01 and *** = p < 0.001.

TCF4 is a key mediator of the Wnt-signaling pathway which is mutationally activated in ~85% of CRCs [4]. Previously, in vivo TCF4 binding domains have been identified in the Ls174T CRC cell line using a ChIP-on-chip approach [16]. Interestingly, this study indicated that TCF4 binds just upstream of the TSS of the long *OSBPL1A *variant as well as upstream of both *TRAK1 *TSSs. This suggests that TCF4 is involved in regulating the TSS usage of these genes. To determine the impact of altered Wnt signaling (TCF4 activity) on the expression of *OSBPL1A *and *TRAK1 *isoforms, overexpression of a dominant negative form of TCF4 (dnTCF4) was used to interrupt the TCF4 mediated part of the Wnt-pathway in the two CRC cell lines, DLD1 and Ls174T [17]. The ratio between the long and the short isoform of *OSBPL1A *was significantly changed in both the DLD1 (p = 6.5 × 10^-4^) and the Ls174T (p = 5 × 10^-6^) cell lines when dnTCF4 was overexpressed (Figure 3A), mainly due to downregulation of the long variant. Interestingly, overexpression of a dominant negative form of TCF1, another transcription factor involved in mediating Wnt signaling, did not alter the ratio between the two *OSBPL1A *isoforms (Figure 3A). Although dnTCF4 targets the same sequence elements as TCF4, it cannot be ruled out that the observed expression changes could be unexpected artifacts of the dnTCF4 overexpression rather than disrupted Wnt-signaling. To address this caveat, we antagonized the signaling cascade upstream of TCF4 (in DLD1 cells) by siRNA mediated knock down of β-catenin, the key regulator of Wnt signaling. As for the dnTCF4 overexpression experiment, this led to a significant change (p = 2.9 × 10^-5^) in the ratio between the two *OSBPL1A *isoforms (with the same direction and magnitude, Figure 3B). This indicated that the Wnt pathway is truly involved in regulating the TSS usage of *OSBPL1A*.

For *TRAK1*, the ratio between the two isoforms shifted significantly, either when dnTCF1 or dnTCF4 was overexpressed (Figure 3C). Surprisingly, the most frequently found isoform in normal mucosa samples was the most infrequent when the Wnt-pathway was disrupted by the overexpression of dnTCF1 or dnTCF4. No change in the isoform ratio was seen when β-catenin was downregulated by siRNA (Figure 3D), implying that the change in *TRAK1 *ratio observed was executed in a β-catenin independent manner.

### Immunohistochemical staining of TCF12 and OSBPL1A in matched normal and tumor tissues

**Figure 4:**
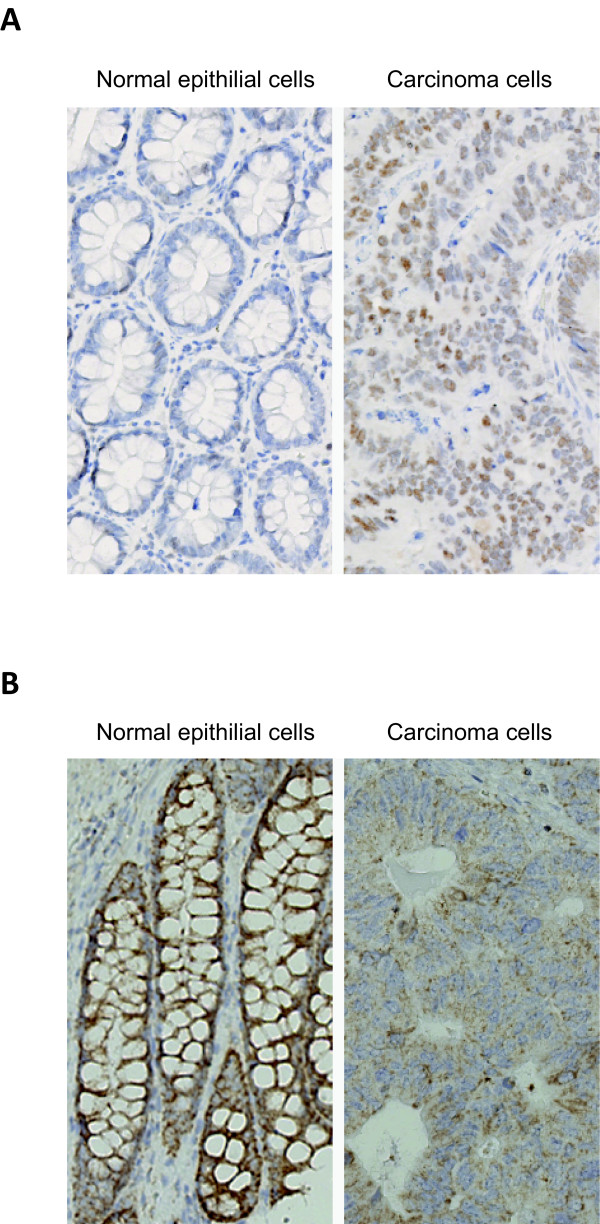
**TCF12 and OSBPL1A expression detected by immunohistochemistry**. A, TCF12 expression detected by IHC in normal epithelial cells and adenocarcinoma cells. B, OSBPL1A expression detected by immunohistochemistry in normal epithelial cells and adenocarcinoma cells. All images are × 10.

To address whether the change in mRNA isoforms had impact on protein expression, immunohistochemical (IHC) analysis of TCF12 was performed on a tissue microarray (TMA) containing 50 normal mucosa and 51 matched adenocarcinoma samples with an antibody specific for an epitope present in both the long and short variant. The IHC revealed a significant increase in both the nuclear staining (p < 0.001, Fishers exact test) and in the percentage of stained cell (p <0.001, Fishers exact test) in cancer compared to normal samples (Figure 4A). This could be explained by the *de novo *synthesis of the short TCF12 isoform in a subset of cancers, as the mRNA encoding the long variant was expressed at equivalent levels in normal and cancer samples. However, this may also reflect other different properties such as a difference in stability of the two isoforms. We observed only very few stained cells in the cancer stroma, corresponding to the weak expression of the short TCF12 mRNA isoform detected in LCM tumor stroma samples.

OSBPL1A IHC staining was performed on the TMA described above with an antibody targeting a peptide found in both transcript variants. This showed a significantly higher cytoplasmic staining intensity in normal epithelium compared to cancer cells (p < 0.001, Fishers exact test), whereas no difference in the percentage of stained cells was observed due to the pervasive expression of OSBPL1A in both normal and cancer tissue (Figure 4B). This was in agreement with our mRNA expression levels as the short OSBPL1A variant is downregulated in cancer samples, whereas the long OSBPL1A variant was expressed at similar levels in the normal and tumor samples.

### TCF12 protein expression is associated with recurrence-free survival

**Figure 5:**
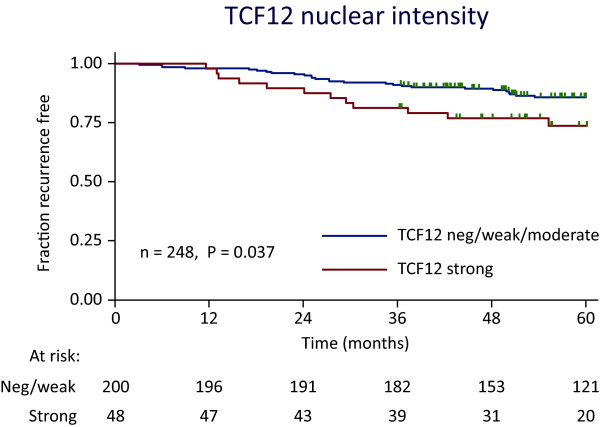
**Kaplan-Meier curves for recurrence-free survival according to TCF12 staining intensity**. IHC staining intensity of TCF12 on a colorectal stage II TMA was used to generate Kaplan-Meier curves for recurrence free survival. Intensity scores were divided in negative/weak/moderate versus strong staining intensity. The log-rank test was used to generate the p-value by comparing the survival curves. Number at risk are listed below the plot and censored samples are indicated with a vertical bar.

To investigate the association between recurrence-free survival and TCF12 or OSBPL1A expression, we used a TMA containing biopsies from 268 stage II adenocarcinomas. Both the intensity of TCF12 and OSBPL1A and the percentage of stained cells were tested for association to recurrence-free survival. A very good inter-observer agreement was seen for all tested IHCs (kappa scores ranging from 0.74-0.83), and we found that TCF12 staining intensity was significantly associated with progression-free survival (log rank test p = 0.037) when data was dichotomized with the respect to strong intensity samples versus negative/weak/moderate intensity samples. Kaplan-Meier curves for TCF12 staining intensity (Figure 5) showed an increased recurrence rate for patients with biopsies that have a strong TCF12 staining. No significant association to recurrence-free survival was observed for the two OSBPL1A IHC staining parameters or the percentage of cells positive for TCF12. Univariate Cox regression analysis was implemented to examine the influence of each of the clinical parameters on progression-free survival, and we found TCF12 nuclear intensity to be significant (p = 0.04)(Table 1). Finally, multivariate analysis was performed including TCF12 nuclear intensity and the number of lymph nodes examined, and we found TCF12 nuclear intensity to be independently associated with progression-free survival (Table 1).

**Table 1:** Univariate and multivariate Cox regression analysis for progression free survival

		Univariate analysis			Multivariate analysis		
**Factor**	**Patients (n = 248)**	**HR**	**95% CI**	**p-val**	**HR**	**95% CI**	**p-val**

TCF12 expression							
Neg/weak	200	1	Reference		1	Reference	
Strong	48	2.03	1.03-4.01	**0.04**	2.24	1.17-4.73	**0.02**
							
Sex							
Female	120	1	Reference				
Male	128	1.82	0.95-3.51	0.07			
							
Age at surgery							
< = 71	128	1	Reference				
>71	120	1.26	0.67-2.38	0.46			
							
Tumour location							
Colon	158	1	Reference				
Rectum	90	1.11	0.57-2.08	0.80			
							
T-stage							
pT3	226	1	Reference				
pT4	22	0.81	0.27-2.80	0.74			
							
Tumor size^a^							
<50 mm	135	1	Reference				
>50 mm	112	0.70	0.35-1.30	0.24			
							
Histological type^a^							
Adenocarcinoma	217	1	Reference				
Mucinous	27	1.18	0.46-4.88	0.50			
							
Number of nodes^a^							
< = 10	119	1	Reference		1	Reference	
>10	119	2.01	1.00-4.04	0.05	2.10	1.00-4.03	0.05

HR = hazard ratio; 95% CI = 95% confidence interval.

^a^Patients with missing values were excluded from the analysis.

### *In silico *protein predictions

The potential structural and functional differences between the differentially expressed isoforms were analyzed using various *in silico *protein function and structure prediction tools, regulatory elements in untranslated regions (UTR) prediction tools along with already reported experimental data [18-24]. The results are summarized in Table 2, where the number of amino acids unique to the long or short variants is listed along with which protein domains are encoded by the unique amino acids, the presence of leader peptides and 5'UTR domains. Notably, significant functional differences are predicted between the long or short isoforms of TCF12, OSBPL1A, TRAK1, CHEK1, ANK3, LMO7 and SCIN indicating that the observed tumor associated changes in TSS usage probably have an impact on the growth properties of the neoplastic cells.

**Table 2:** Protein and 5' UTR *in silico *predictions

	**Unique AA^a^**		**Protein domains**		**Signal peptide**		**Glucosylation and phosphorylation**		**5'UTR**	
Gene	Long	Short	Long	Short	Long	Short	Long	Short	Long	Short
TCF12	193	23			Yes	Yes	10 glyco, 27 phos			
OSBPL1A	513		3 Ank^b^,Pleck^c^		No	No	31 phos			
TRAK1	96	38			Yes	No				
CHEK1	0				No	No			uORF^d^	
ANK3	872	6	20 Ank^b^		No	No	3 glyco, 33 phos			
UGP2	11				No	No				uORF^d^
LMO7	285		Calponin		Yes	No	21 phos		IRES^e^	
ACSL5	56				No	Yes				
SCIN	247		2 Gelsolin		No	No				uORF^d^

^a^AA = Amino acids

^b^Ank = Ankyrin repeats

^c^Pleck = Pleckstrin

^d^uORF = Upstream open reading frame

^e^IRES = Internal ribosome entry site

## Discussion

This study reports the results of a genome-wide screening for alternative TSS usage between normal and neoplastic colorectal samples. In total, we identified nine genes with alternative TSS usage and validated them in an independent cohort. A subset of the candidates was validated by qRT-PCR on the RNA used for array analysis and on RNA from LCM samples. TF binding analysis suggested that p53 and SOX9 might be responsible for altering the TSS use as they are both upregulated in cancer and have enriched binding sites in the promoters associated with the TSS preferred by the neoplastic cells. Disruption of Wnt signaling using two different CRC cell lines further showed that perturbed Wnt-signaling may also play a role in regulating alternative TSS usage of *OSBPL1A *and *TRAK1*, maybe through SOX9 which is known to be a Wnt-regulated TF [15].

The vast majority of scientific reports commonly refer to individual genes as one entity encoding a predominant transcript. This is a drastic oversimplification considering that experimental evidence supports the existence of at least two functional promoters for > 50% of all genes [25,26], and, furthermore, it has the unfortunate consequence that potentially important transcriptional variants may be ignored. Such transcript diversity is generated by various mechanisms such as alternative splicing, alternative TSS usage and alternative polyadenylation usage, which generate families of transcripts from a single gene locus. Additionally, the use of DNA methylation appears to contribute to differential usage of alternative promoters in multiple tissue types [27] as well as in cancer [28]. We have previously detected cancer specific alternative splicing in colorectal, prostate and bladder cancer [29], and also shown that disruption of Wnt signaling can change the splicing pattern in colorectal, lung and gastric cancer [6]. The findings in this paper extend these previous observations by providing specific examples of transcript isoforms that arise through tumor-specific alternative TSS usage that occur not only in colorectal, but also in a wide range of other cancers. We have not addressed potential alternative splicing of *TCF12, OSBPLA1 *and *TRAK1 *in this study, but have shown that the exon expression pattern and qPCR validation is consistent with alternative TSS. In *TCF12 *and *OSBPL1A*, the expression follows the pattern of already described short and long transcript isoforms, and for alternative splicing to explain this pattern, novel extreme 5' start exons would have to be included in the transcript of both genes followed by alternative splicing of at least eight otherwise constituve spliced exons, a scenario we consider highly unlikely.

For the transcriptional changes to impact tumor cell growth, it is crucial that they translate into protein changes. We used two different strategies to address this issue. First, IHC analysis showed a significant difference in the total protein level of TCF12 and OSBPL1A between normal and cancer samples consistent with the mRNA expression pattern we observed. Furthermore, we found a correlation between the total TCF12 protein expression level and progression free survival. We did not perform IHC analysis of TRAK1 as a recent study already reported that elevated levels of TRAK1 are associated with poor prognosis in CRC patients [30]. Second, *in silico *analysis was used to predict the impact of an altered TSS on protein function, and we found that OSBPL1A has 31 phosphorylation sites and protein interaction domains that are unique to the long isoform, indicating a loss of function for the short isoforms. The long TCF12 variant encodes 10 glycosylation and Yin-Yang sites along with 27 phosphorylation sites not present in the short isoform. However, both isoforms contain DNA binding and dimerization domains, indicating separate functions of the isoforms, and not a simple dominant negative function of the short isoform. Indeed, both the long and short isoform of the TCF12 have previously been described as having distinct functions in thymocyte development [31,32]. Deregulation of *Tcf12 *has recently been causally implicated in CRC in a DNA transposon based forward genetic screen in a mouse model. Interestingly, in this study, *Tcf12 *was the fourth most commonly mutated gene locus (*Apc *being the most commonly mutated gene) [33]. This clearly indicates that *TCF12 *could have an important effect in CRC development. *In silico *predictions for the remaining candidate genes indicated diverse functional differences such as altered protein localization, changes in protein-protein interaction domains and differences in translational regulation and efficiency.

The selective use of alternative TSSs has been observed in different cell types, tissue types and developmental stages [8], however, the mechanism regulating the alternative TSS usage is often less well described. Aberrant TSS usage has also been linked to cancer, and *in vitro *studies of hypoxia in a CRC cell line revealed hundreds of genes with altered TSSs [34]. To elaborate on the possible mechanisms regulating the alternative TSS usage in CRC, we combined TF binding site enrichment analysis and TF expression analysis and showed that several TFs were dysregulated in tumors along with having enriched binding sites in the TSS regions. Furthermore, we used cell line models to demonstrate that abrogation of Wnt signalling leads to altered TSS usage for *OSBPL1A *and *TRAK1*, showing that the major pathway in CRC development could be a key regulator of TSS usage.

## Conclusions

The use of alternative TSSs is a widespread phenomenon in human genes, and by using exon array analysis, we identified nine genes with differential expression of isoforms between normal and tumor samples arising from differential TSS use. The changes observed for *CHEK1, OSBPL1A *and *TCF12 *were confirmed in several other cancer types indicating that the change in TSS usage is a general mechanism in cancer biology. The shift in *OSBPL1A *isoform ratio was experimentally shown to be regulated by the Wnt pathway, which is deregulated not only in CRC, but also in many other cancer types. A *TCF12 *short variant was found to be *de novo *synthesized in colorectal tumors, and TCF12 protein level was shown to be associated with progression free survival, further underlining the potential importance of alternative TSS usage in cancer development.

## Methods

### Clinical samples

One hundred and eight colorectal patient biopsies comprising 24 adjacent normal mucosa samples, 49 MSS adenomas (16 tubular, 12 tubulovillous and 21 villous), 30 MSS carcinomas (one stage I, 16 stage II and 13 stage III) and five MSI carcinomas (four stage II and one stage III) were examined (see Additional file 4 for a summary of the histopathological characteristics). All carcinomas were classified according to the WHO/UICC-TNM staging system. Immediately after surgery or polypectomy, the biopsies were embedded in Tissue-Tek O.C.T. Compound (Sakura Finetek), snap-frozen in liquid nitrogen and stored at -80°C. All patients gave informed written consent, and the study was approved by the Central Denmark Region Committee on Biomedical Research Ethics according to the Helsinki Declaration.

For recurrence free survival analysis, IHC was performed on a human colorectal TMA containing 268 biopsies from stage II adenocarcinomas. The fraction of patients with recurrence (distant metastasis excluding carcinosis) was 42 (16%). The median duration of follow-up for the non-recurrence group was 1709 days (range 1099-1825 days), and for the recurrence group 770 days (range 95-1681). Before progression free survival analysis, 20 biopsies were excluded because the core was missing or lacked tumor material, leaving 248 samples for the analysis. A TMA containing 51 stage II adenocarcinomas and 50 normal mucosa samples was used to assess the expression of TCF12 and OSBPL1A.

### RNA preparation and Human Exon 1.0 ST Array labeling

A Hematoxylin and Eosin stained cryostat section from all samples was used to evaluate tissue composition and when necessary macroscopic trimming was used to enrich the fraction of tumor cells to ensure a minimum of 60% neoplastic cells (median 85% (60%-90%)). Total RNA was isolated from serial cryo-sections using the RNeasy Mini elute kit (Qiagen). Quality of the RNA was evaluated on the 2100 Bioanalyzer (Agilent), the median RNA Integrity Number (RIN) was 9.1 (6-10), and the median 28S/18S ribosomal peak ratio was 1.8 (1.1-3.1).

One hundred ng of total RNA was labeled according to the GeneChip Whole Transcript (WT) Sense Target Labeling Assay (Affymetrix, Inc., Santa Clara, CA) and hybridized to Human Exon 1.0 ST Arrays (Affymetrix, Inc., Santa Clara, CA) overnight. Scanning was performed in an Affymetrix GCS 3000 7G scanner.

### Exon array data analysis

Quantile normalization of exon array data and all further analysis was performed in the GeneSpring GX10 software (Agilent) using ExonRMA16 with core transcripts (17881 transcripts). For stabilization of variance, 16 was added to expression values before log2 transformation, which resulted in a minimum value of four in the log2 transformed dataset. Differential gene expression analysis was performed on transcript values based on core probe sets using Benjamini-Hochberg corrected unpaired t-tests. Alternative TSS analysis was limited to genes in the hg18 Refseq database containing two or more transcription start sites and at least one protein-coding isoform (2176 genes). To have probe sets in all exons of these genes, both core and extended probe sets of the exon array [35] were included in analysis of alternative TSS. Only genes for which more than 50% of the probe sets were expressed above background in at least half of both the normal and the tumor samples were included in the TSS analysis. Genes with potential alternative TSS or alternative splicing were identified using a multivariate ANOVA analysis (splicing ANOVA) and 663 genes had a splicing ANOVA p-value < 0.05 (Benjamini-Hochberg corrected). More stringent filtration on the p-value (<10^-6^, Benjamini-Hochberg corrected) and the log2 splicing index (SI = probe set intensity/transcript expression value) SI > 0.5 or SI < -0.5 resulted in 156 candidate genes. To identify genes expressing isoforms with alternative TSSs, the expression data of the candidate genes were visualized in a genomic context using the UCSC genome browser [36]. This manual curation identified nine candidate genes with apparent alternative TSS usage, which were further analyzed in two independent validation sample sets, and it was required that candidate genes were found to be significant in at least one of the validation sets (splicing ANOVA p-value (< 0.05) and SI (> 0.5 or SI < -0.5)). The student's t-test was used when comparing two sample groups, except for the analysis of paired samples were a paired t-test was used.

### Quantitative real-time reverse-transcription PCR

One μg of total RNA was converted to cDNA using a mixture of oligo(dT) and random nonamer primers and Superscript II Reverse Transcriptase (Invitrogen). Quantitative real-time reverse-transcription PCR (qRT-PCR) was performed in triplicates on a 7500 Fast or 7900HT Real-Time PCR System (Applied Biosystems). See Additional file 5 for the sequences of the primers used. Normalization was performed with UBC as previously described [37].

### Laser capture microdissection

Laser capture microdissection was performed on cryosections from paired cancer and adjacent normal colon mucosa biopsies from six patients (two stage II and four stage III). Briefly, the sections were fixed in 95% EtOH for 120 sec, followed by 15 sec of staining in Arcturus Histogene Staining Solution (DFA Instruments), dehydrated in 95% EtOH (30 sec) and 100% EtOH (120 sec) before a final treatment in xylene for 120 sec. After drying of slides, epithelial and stromal cells were captured on individual caps using the Veritas 704 apparatus (Arcturus). Captured material was incubated with RLT buffer (Qiagen) for 20 minutes at room temperature in the presence of 30 mM β-mercaptoethanol, and, subsequently, RNA was extracted using RNeasy MinElute spin columns (Qiagen). Five ng RNA was used for cDNA synthesis using the Ovation PicoSL WTA System (NuGEN) followed by qRT-PCR as described above.

### TMA staining and scoring

Staining of the TMAs was performed with the following antibodies; Anti-TCF12 (Catalog Number: 14419-1-AP, ProteinTech) in a 1:250 dilution and anti-OSBPL1A (Catalog Number: 18-202-335518, Genway Biotech) in a 1:400 dilution and indirect staining was used as previously described [38].

The TMAs were scored by two independent investigators using the VIS software (Visiopharm). The intensity of the staining was scored within the following categories (negative, 0; weak, 1; moderate, 2 and strong, 3) and the fraction of positive cancer cells (negative, 0; less than half, 1; 51-80%, 2 and >80%, 3) was evaluated independently by two investigators. The agreement between the investigators was evaluated by Kappa statistics. STATA 9.2 software (StataCorp) was used to perform univariate analysis using the log rank test or the Cox proportional hazards model, for the Fishers exact test and for generating Kaplan-Meier plots.

### Wnt-pathway model system

The colon cancer cell lines (Ls174T and DLD1) stably expressing inducible dominant-negative (dn)TCF1 or dnTCF4 were a kind gift from Dr. Hans Clevers (The Hubrecht Laboratory, The Netherlands), and have previously been described [39]. β-catenin knockdown was performed in DLD1 cells by transfecting with 20 nM siRNA targeting β-catenin (Dharmacon) or 20 nM scrambled nontargeting siRNA. Transfections were carried out using Lipofectamine 2000 (Invitrogen). RNeasy (Qiagen) was used for RNA extraction and random nonamer primers were used for cDNA synthesis. qRT-PCR was performed as described above.

### Transcription factor binding analysis

For each of the selected genes, 300 bp upstream and 100 bp downstream of the transcription start site were analyzed for transcription factor binding sites. Using the TOUCAN2 program [14], the regions were scanned for known binding domains from the Transfac Public v7 vertebrate database. Domains overrepresented relative to the Eukaryotic Promoter Database [40] were selected, and tumor and normal samples were compared.

### Additional Human Exon 1.0 ST datasets

The external colon cancer validation dataset was downloaded from http://www.affymetrix.com and consisted of 18 paired samples of adenocarcinoma and adjacent normal tissue [35]. The internal independent colon cancer validation set consisted of nine adjacent normal biopsies, six tubular adenomas, 13 MSS cancer and 6 MSI cancer samples and has previously been described [29]. The bladder cancer dataset consisted of 11 samples of normal epithelium, 12 T1tumors and 12 T2-4 tumors [29]. Five additional datasets covering brain gliomas (GSE9385), gastric (GSE13195), lung (GSE12236), liver (GSE12941) and prostate cancer (GSE21034) were downloaded from the Gene Expression Omnibus. The brain glioma dataset contained 26 glioblastomas, 22 oligodendrogliomas and 6 control brain samples [41]. The gastric cancer dataset consisted of 44 paired samples of adenocarcinoma and adjacent normal tissue. The lung cancer dataset consisted of 40 paired normal and lung adenocarcinomas [42]. The liver cancer dataset contained 20 paired samples of hepatocellular carcinoma and adjacent nontumorous liver [43], and the prostate cancer dataset consisted of 29 normal adjacent benign samples, 131 primary tumors and 19 metastases [44].

### Bioinformatics analysis of differentially expressed protein features and mRNA regulatory elements

First, the peptides corresponding to the unique sequences of the different isoforms were aligned against the Protein Data Bank database by PSIBLAST [45]. Then FeatureMap3D [46], PyMOL (Molecular Graphics System, Delano, Scientific system, LLC, Palo Alto, CA), and scripts created for the analysis were used to analyze and visualize the structures and/or conserved domains. Secondary structures were predicted by PSIpred [47]. Hydrophobicity plots were based on Kyte-Doolittle and Hopp-Woods scales to predict potential hydrophilic regions most likely exposed on the protein surface. NetPhos [18] was used to predict phosphorylation sites. Furthermore, signal peptide cleavage site prediction was performed [20] along with prediction of propeptide cleavage sites, Yin-Yang sites and N-glycosylation and O-glycosylation sites [48]. Protein functions that potentially change between alternative isoforms were compared with ProtFun [49]. Regulatory RNA elements in UTRs related to transcriptional and translational regulation were predicted by RegRNA [22,50,51].

## Authors' contributions

KT carried out the exon array analysis, the qRT-PCR validation, participated in the TMA scoring and wrote the manuscript. TS performed the Wnt model experiments. BØ carried out the selection of samples and extraction of RNA. MHR carried out the laser capture microdissection and cDNA synthesis and contributed to writing the manuscript. SV implemented the TF binding analysis. KW performed all *in silico *protein predictions. KQH stained the tissue sections and participated in the TMA scoring. PL and JSP assisted with the data analysis. AE constructed the TMAs. FM participated in the protein analysis. KL and CW created the refseq gene lists and assisted with data analysis. SL provided patient material and clinical data. LD assisted in exon array analysis. TFO and CLA participated in the design and coordination and participated in the writing of the manuscript. All authors read and approved the final manuscript.

## Supplementary Material

Additional file 1 Expression of *TCF12, OSBPL1A *and *TRAK1 *in paired normal and tumor samples Click here for file

Additional file 2 qRT-PCR of laser capture microdissected samples Click here for file

Additional file 3 *OSBPL1A *and *TCF12 *isoform expression in multiple cancer types Click here for file

Additional file 4 Table with summary of the histopathological characteristics Click here for file

Additional file 5 Sequence of primers used for qRT-PCR Click here for file
