# Supplementary material for: Tumor-specific usage of alternative transcription start sites in colorectal cancer identified by genome-wide exon array analysis
Source: BMC Genomics. 2011 Oct 14;12:505. doi: 10.1186/1471-2164-12-505 (PMC3208247; doi:10.1186/1471-2164-12-505)
Supplement: Additional file 1 — Expression of TCF12, OSBPL1A and TRAK1 in paired normal and tumor samples [file 1471-2164-12-505-S1.PDF]

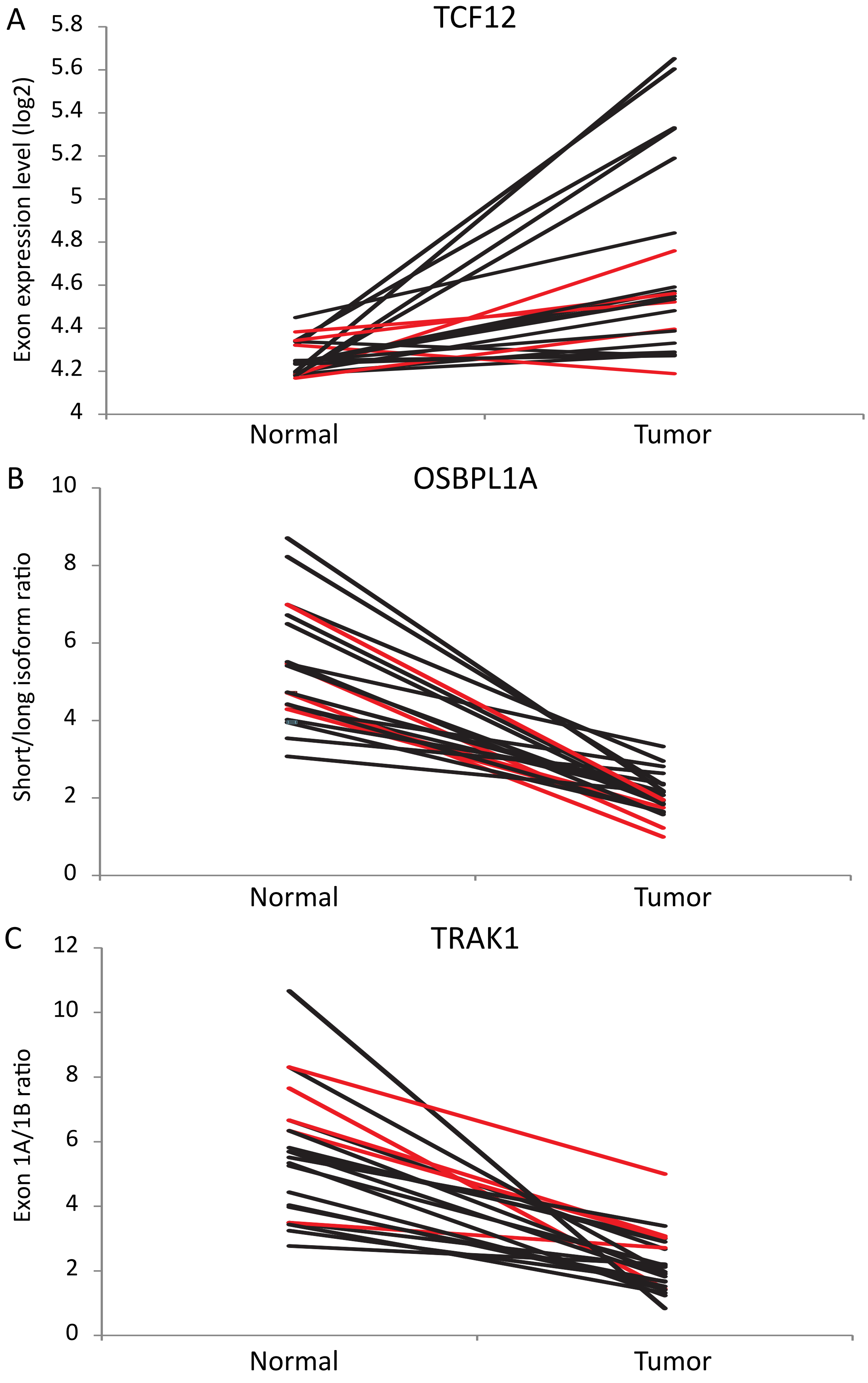

Additional file 1: Expression of *TCF12, OSBPL1A* and *TRAK1* in paired normal and tumor samples
Exon array expression data from eighten pairs of normal and adenoma samples (black lines), and five pairs of normal and cancer biopsies (red lines) were available. A, *TCF12* exon 1B expression. B, *OSBPL1A* isoform ratio (short/long). C, *TRAK1* isoform ratio (1A/1B)
