# Supplementary material for: Tumor-specific usage of alternative transcription start sites in colorectal cancer identified by genome-wide exon array analysis
Source: BMC Genomics. 2011 Oct 14;12:505. doi: 10.1186/1471-2164-12-505 (PMC3208247; doi:10.1186/1471-2164-12-505)
Supplement: Additional file 2 — qRT-PCR of laser capture microdissected samples [file 1471-2164-12-505-S2.PDF]

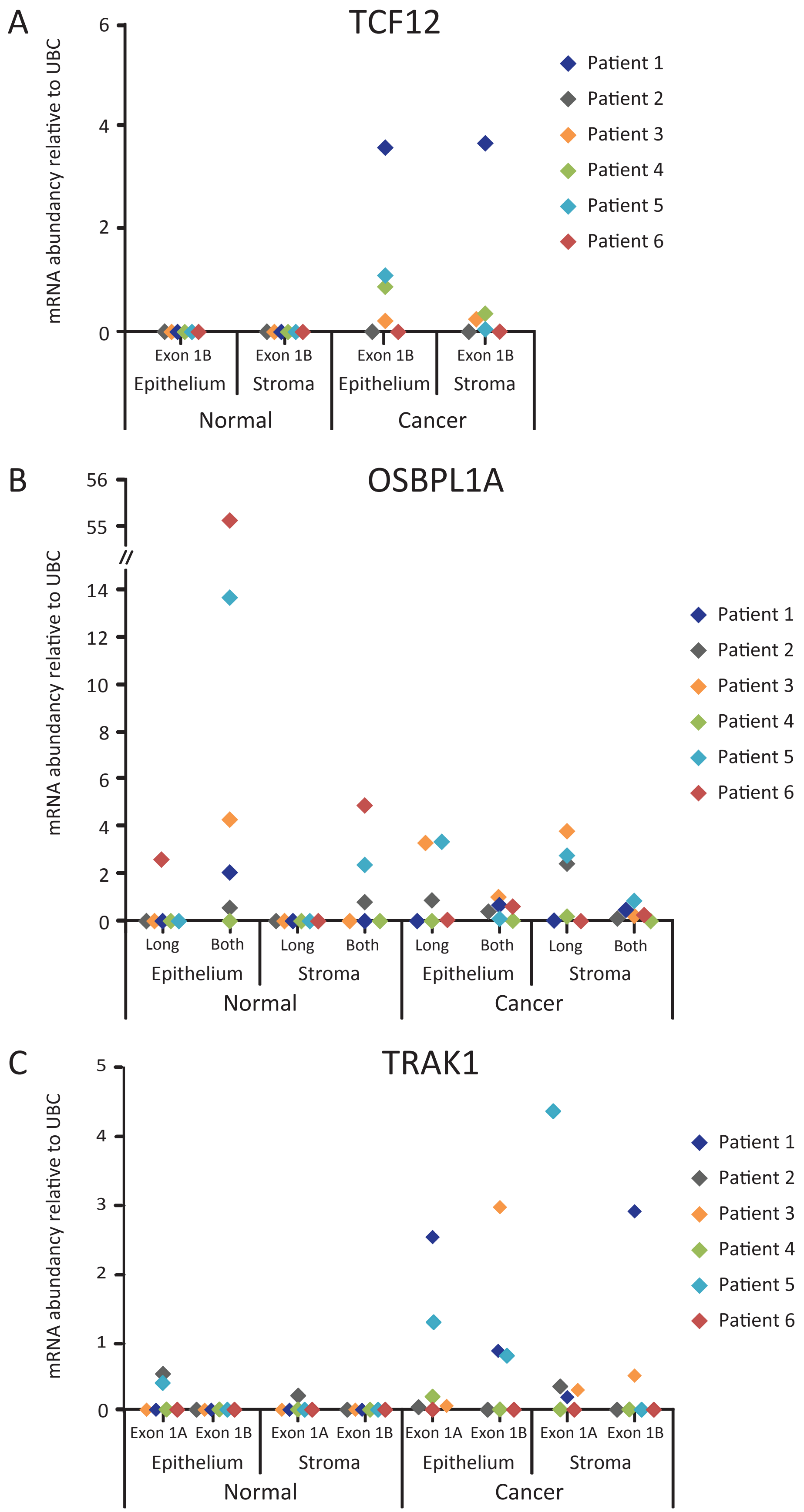


**Additional file 2: qRT-PCR analysis of laser capture microdissected samples**
Cancer and adjacent normal biopsies from six patients were laser capture microdissected to give four fractions from each patient (normal epithelial cells, stroma from normal biopsies, colorectal cancer cells and cancer derived stroma). A, expression of the *TCF12* alternative start exon (1B) in the four cell fractions. B, expression of the long *OSBPL1A* isoform (Long) and the expression of both isoforms (Both) measured by primers specific to exons present in both isoforms. C, expression of *TRAK1* exon 1A and exon 1B.
