# Supplementary material for: Tumor-specific usage of alternative transcription start sites in colorectal cancer identified by genome-wide exon array analysis
Source: BMC Genomics. 2011 Oct 14;12:505. doi: 10.1186/1471-2164-12-505 (PMC3208247; doi:10.1186/1471-2164-12-505)
Supplement: Additional file 3 — OSBPL1A and TCF12 isoform expression in multiple cancer types [file 1471-2164-12-505-S3.PDF]

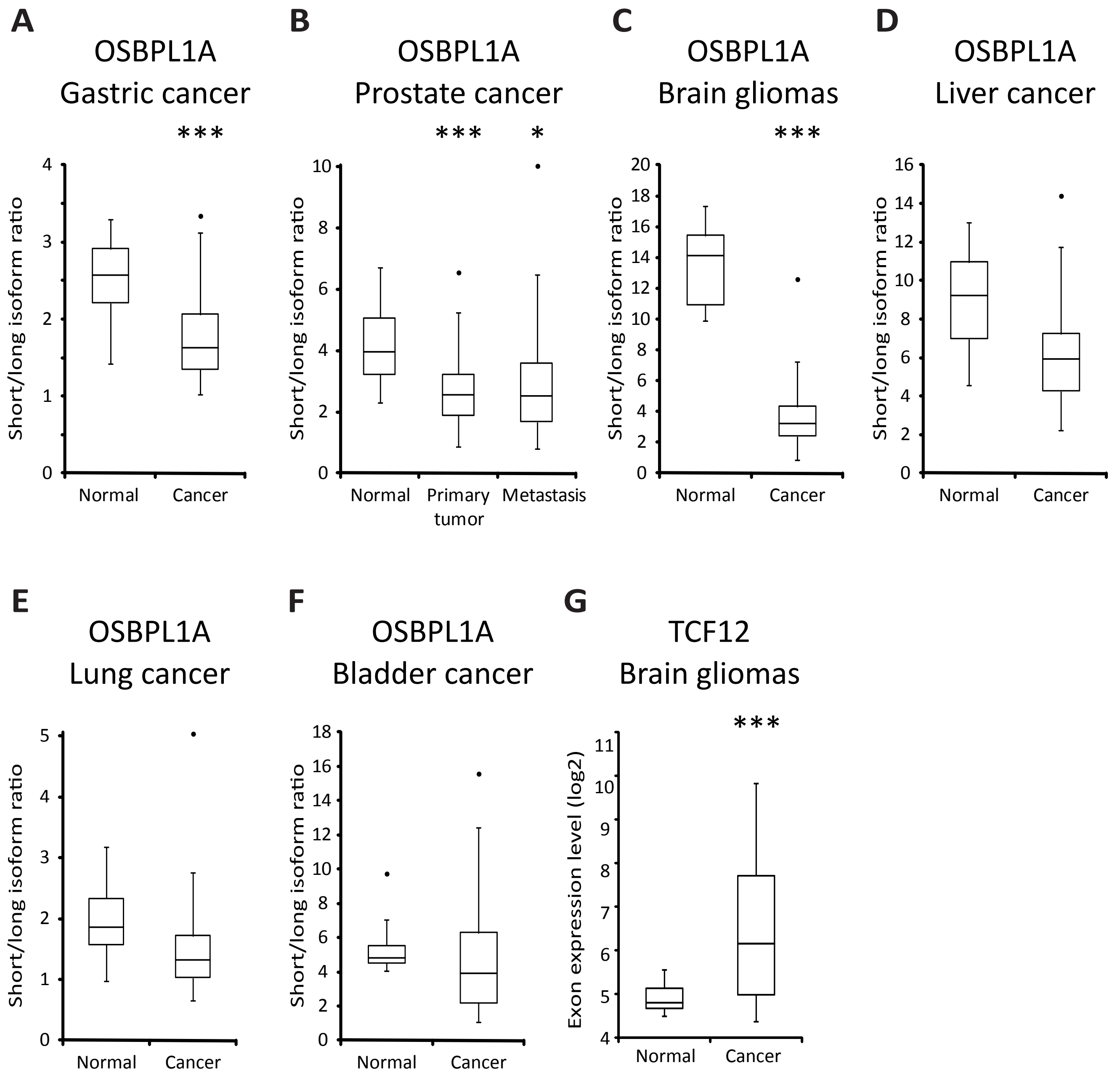

Additional file 3: *OSBPL1A* and *TCF12* isoform expression in multiple cancer types

A, *OSBPL1A* isoform expression ratio in normal gastric and gastric cancer samples.B, *OSBPL1A* expression in prostate normal, prostate cancer and metastasis from prostate cancer patients, C, *OSBPL1A* expression in normal brain and brain gliomas, D, *OSBPL1A* expression in liver normal and cancer, E, *OSBPL1A* expression in lung normal and cancer, F, *OSBPL1A* expression in bladder normal and cancer samples and G, *TCF12* expression in normal brain and brain gliomas.
* = $p < 0.05$, ** = $p < 0.01$ and *** = $p < 0.001$.
